# Combinatorial Effects of Transposable Elements on Gene Expression and Phenotypic Robustness in *Drosophila melanogaster* Development

**DOI:** 10.1534/g3.113.006791

**Published:** 2013-09-01

**Authors:** Alexa W. Clemmons, Steven A. Wasserman

**Affiliations:** Section of Cell & Developmental Biology, University of California San Diego, La Jolla, California 92093-0349

**Keywords:** transcriptional fluctuation, stochasticity, transposable elements, embryonic patterning, phenotypic variability

## Abstract

Embryonic patterning displays remarkable consistency from individual to individual despite frequent environmental perturbations and diverse genetic contexts. Stochastic influences on the cellular environment may cause transcription rates to fluctuate, but these fluctuations rarely lead to developmental defects or disease. Here we characterize a set of recessive alleles of the Toll pathway component *tube* that destabilize embryonic dorsoventral patterning in *Drosophila melanogaster*. Females bearing these *tube* alleles generate embryos of an unusually wide range of dorsalized phenotypes, with the distributions across this range being unique for each allele. We determine that the mutant lines have in common a retrotransposon insertion upstream of the *tube* transcription start site. Genetic and molecular approaches demonstrate that this insertion dramatically reduces maternal expression of *tube*, thereby uncovering the inherent variability in gene expression. We further find that additional transposable element insertions near the *tube* gene synergistically enhance the phenotype caused by the sensitizing upstream insertion. These studies document how phenotypic variability can arise from normally occurring fluctuations around reduced mean expression and illustrate the contribution of transposons, individually and combinatorially, to such a state.

Gene expression often varies over time within a single cell or among cells of the same tissue. This variation exists, in part, because stochastic forces influence transcription (Chubb and Liverpool 2010; Kaern *et al.* 2005; Lehner and Kaneko 2011). The source of stochasticity itself varies and includes both the sporadic fluctuations in local transcription factor abundance and the dynamic nature of chromatin. Cells generally buffer this transcriptional noise, avoiding any detrimental effects and thus displaying a property termed phenotypic robustness (Flatt 2005; Waddington 1942). Developmental biologists have long been intrigued by the way wild-type organisms achieve robust patterning by dampening the effects of environmental, genetic, and stochastic perturbations (Arias and Hayward 2006; Houchmandzadeh *et al.* 2002; Porcher and Dostatni 2010).

Survival requires maintaining transcript levels of essential genes above a threshold value. Gene expression at levels significantly above the threshold is one potential means of lessening the effects of noisy gene expression; expression levels swing back and forth around an average, but the entire range lies above the threshold. In this model, the detrimental effects of stochastic forces on phenotype in a wild-type organism are minimized.

For the vast majority of *Drosophila melanogaster* genes, changing dosage does not affect survival, as demonstrated by investigations of segmental aneuploids (flies in which particular autosomal regions of the genome are present in only a single copy or in three copies). In their landmark investigation of the *D*. *melanogaster* genome, Lindsley *et al.* (1972) demonstrated the existence of at most 20 loci, and more likely just one, that are haploinsufficient for viability. This finding, together with subsequent studies, revealed that nearly all genes are normally expressed at levels greater than that required for survival, consistent with the idea that surplus gene expression contributes to phenotypic robustness.

On the basis of the aforementioned model, one would predict that fluctuations produced by stochastic forces would be revealed if an additional influence, such as a mutation, reduced the average expression level of a gene to near or below the threshold. The phenotype would then vary with changes in expression, essentially becoming a readout of the probabilistic nature of underlying molecular interactions.

With the exception of temperature-sensitive and other conditional missense alleles, mutations that disrupt developmental patterning typically result in a consistent and relatively narrow phenotypic range. Occasionally, however, phenotypic hypervariability emerges (Raj *et al.* 2010). What distinguishes these rare cases? It may be that some processes, like transcription, are more sensitive to perturbations than others. To investigate this phenotypic phenomenon, we focused our attention on a set of mutations exhibiting hypervariable disruption of Toll signaling.

In the *D. melanogaster* embryo, the Toll pathway establishes dorsoventral polarity. Females bearing mutations that block Toll signaling produce dorsalized embryos, with the severity of dorsalization corresponding to the extent of reduction in signaling (Supporting Information, Figure S1 and Anderson and Nüsslein-Volhard 1984; Huang *et al.* 1997). Generally, isogenic females bearing a mutation in a Toll pathway gene produce embryos of a very narrow phenotypic range. The mutations that we have studied, which affect the Toll pathway adaptor protein Tube, instead cause an unusually wide range of phenotypes. We have used these *tube* mutants as a model system to study variable gene expression and phenotypic robustness.

## Materials and Methods

### Fly stocks, site-specific male recombination, precise excision, and cuticle preparation

Alleles *tub^2^*, *Df(3R)XM3*, *tub^R5.6^*, *tub^6^*, *tub^7^*, *tub^8^*, and *tub^9^* have been described previously (Hecht and Anderson 1993; Letsou *et al.* 1991). The *tub^ste^* allele was identified on a *st e* marker chromosome obtained in the 1980s from the K. V. Anderson lab. The wild type (*tub^+^/tub^+^*), unless otherwise noted, was *P{His2Av-EGFP.C}2/SM6a*, obtained from Bloomington Drosophila Stock Center. *Df(3R)XM3* served as *tub^Df^* and *tub^R5.6^* served as *tub^null^* in all experiments, except where otherwise noted. *CG14646^CB-0692-3^* (*CB06923)*, *GS7007*, and *GS13951* were obtained from the Drosophila Genetic Resource Center at the Kyoto Institute of Technology. Site-specific recombination and precise excision were performed with the use of a transposase source from the stock *T(2;3)ap^Xa^*, *ap^Xa^/CyO*, *H[PΔ2-3]HoP2.1*; *Sb* obtained from Bloomington Drosophila Stock Center. Site-specific recombination was conducted as previously described (Chen *et al.* 1998). Precise excision was conducted by generating males bearing the Δ2−3 source and the *tub^9^* chromosome, collecting their female progeny, mating them with wild-type males at 18°, and assaying for increased fecundity. Genomic DNA from potential excisants was amplified via polymerase chain reaction (PCR) and sequenced to identify precise excisants. For all experiments examining dorsalization, cuticles from embryos (1−2 d after fertilization) raised at 25° were prepared and scored as previously described (Wieschaus and Nüsslein-Volhard 1986), unless otherwise noted.

### Survival assays

Survival assays were performed essentially as described previously (Romeo and Lemaitre 2008). Males (2−4 days posteclosion) were stabbed with a needle dipped in a 20% glycerol suspension of purified fungal spores; the fungus used was *Fusarium oxysporum* f. sp. *lycopersici* (obtained from the Fungal Genetics Stock Center). Flies were incubated at 29° for the duration of the experiment. Survival was assayed over 4 d.

### Quantitative real-time (RT)-PCR, sequencing, and 5′ rapid amplication of cDNA ends (5′ RACE)

RNA was prepared using Trizol (Ambion) or RNeasy kit (QIAGEN) from embryos (0−1.5 hr after fertilization) or adult males (2−5 days after eclosion), and first-strand cDNA was synthesized with the SuperScript II kit (Invitrogen). Quantitative RT-PCR was performed on an iQ5 cycler (BioRad) using iQ SYBR Green Supermix (BioRad). Genomic DNA was prepared from adults as described previously (Huang *et al.* 2009). Taq Polymerase with ThermoPol buffer (NEB) and Expand HF kit (Roche) were used to amplify the *tube* transcript region and flanking regions for sequencing. Thermal asymmetric-interlaced (TAIL)-PCR was conducted essentially as described previously (Liu and Whittier 1995), except Phusion (NEB) was used as the polymerase. 5′ RACE was performed using the RLM-RACE kit (Ambion), and Phusion (NEB) was used as the PCR polymerase.

### Immunoblotting

Immunoblotting protocols and rabbit α-Tube serum (1:20,000) have been described previously (Letsou *et al.* 1993; Sun *et al.* 2004). Rabbit α-Diaphanous (1:5,000) was used as a loading control and was previously described (Afshar *et al.* 2000). Secondary antibody was goat α-rabbit IgG-peroxidase (1:10,000; Sigma-Aldrich).

### Statistics

Quantitative RT-PCR data were analyzed by use of a one-way analysis of variance test followed by a Dunnett post-test (GraphPad PRISM 5).

## Results

### Phenotypically variable *tube* alleles

**Figure 1:**
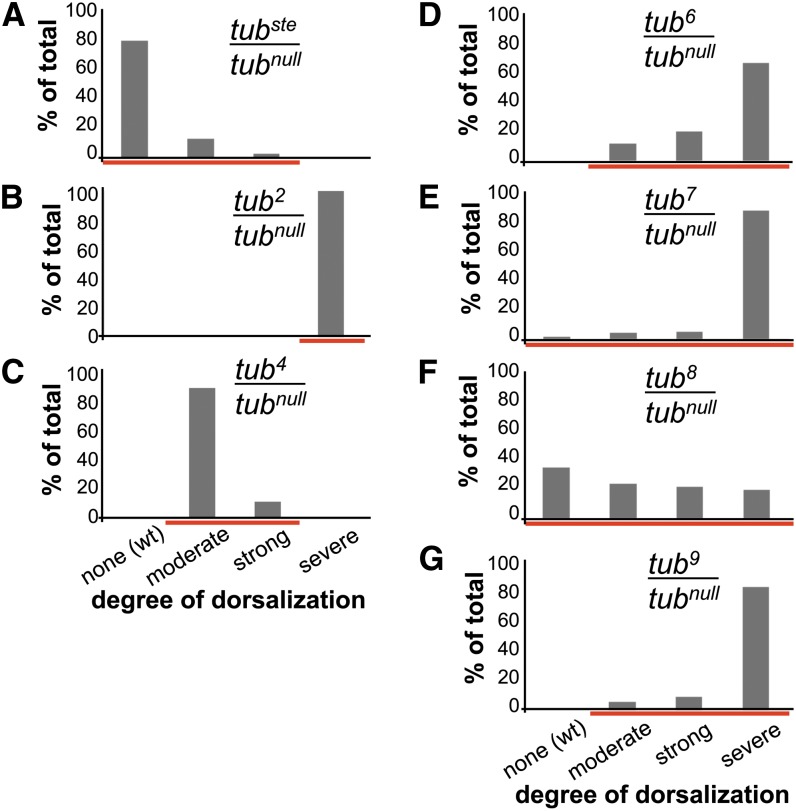
Embryos from *tub^var^/tub^null^* females display a wide range of dorsalized phenotypes compared with conventional alleles, *tub^2^* and *tub^4^*. (A−G) Phenotypic distributions of embryonic cuticles from groups of females of the specified genotype. Analysis of *tub^7^* was performed at 18° and in *trans* to *tub^2^*, a strong hypomorphic allele. Red lines highlight phenotypic range. (A) n = 77, (B) n = 56, (C) n = 34, (D) n = 190, (E) n = 160, (F) n = 213, and (G) n = 197.

The starting point for these studies was the finding that a particular *D. melanogaster* chromosome provides variably reduced *tube* function. Because this *tube* allele was discovered on a marker chromosome containing visible mutations in the genes *scarlet* and *ebony* (*st*, *e*), we named it *tub^ste^*. Females carrying the *tub^ste^* chromosome in *trans* to a *tube* null mutation (*tub^ste^/tub^null^*) produce embryos that span a phenotypic range from strongly dorsalized to wild type (Figure 1A and Figure S1). As stated previously, except in cases of conditional missense alleles, such phenotypic variation is rare. For example, the phenotypes of *tub^2^* and *tub^4^*, which disrupt *tube* function to quite different degrees, are distinct but nevertheless largely invariant (Figure 1, B and C). There is, however, a set of alleles that, like *tub^ste^*, displays an unusually wide phenotypic range. Hecht and Anderson (1993) have reported isolation of four alleles—*tub^6^*, *tub^7^*, *tub^8^*, and *tub^9^*—exhibiting highly variable *tube* function. To compare the phenotypic variation of *tub^ste^* with these *tube* alleles, we analyzed embryonic cuticles. Consistent with the published data, embryos from females carrying any of these alleles in *trans* to a strong or null *tube* mutation exhibit a broad range of phenotypes, with a distinct phenotypic distribution for each allele (Figure 1, D−G). Hereafter, we therefore use the term *tub^var^* to refer to the variable alleles *tub^ste^*, *tub^6^*, *tub^7^*, *tub^8^*, and *tub^9^*.

Phenotypic variability typically arises from heterogeneity in genetic background or the environment. However, when flies are held in a constant environment, a single *tub^var^*/*tub^null^* female, like a population of *tub^var^/tub^null^* females, generates embryos with a range of dorsalization (compare Figure 1 and Figure S2; see also Hecht and Anderson 1993). Thus, the phenotypic range does not reflect variation in genetic background or environment, nor does it reflect paternal genotype because *tube* function in embryonic patterning is strictly maternally contributed (Gerttula *et al.* 1988). Rather, the *tub^var^* chromosomes must provide variable *tube* activity, reflecting an alteration in the production, stability, or activity of the *tube* mRNA or protein.

The observation that single genotypes give rise to widely variable phenotypes suggests a stochastic contribution to *tube* function. One possible source of stochasticity is the effect of cellular fluctuations on the activity or stability of a protein or RNA transcript. However, sequencing revealed that the Tube proteins encoded by four of the five *tub^var^* alleles are wild-type (the *tub^6^* coding sequence has an asparagine in place of aspartic acid at position 106). Moreover, the noncoding portion of the *tube* transcription unit is wild-type for all of the *tub^var^* alleles, making a disruption in mRNA processing or stability very unlikely. What then is the source of phenotypic variation for these *tub^var^* alleles?

### Stochastic variation in *tube* expression

On the basis of these findings, we postulated that the *tub^var^* alleles alter *tube* transcription. In particular, we envision that these mutations reduce mRNA levels below a threshold amount, revealing phenotypic effects of stochastic gene expression fluctuations. To determine if *tub^var^* alleles on average substantially reduce gene expression, we assayed *tube* expression in batches of embryos from *tub^var^/tub^null^* females by both quantitative RT-PCR (qRT-PCR) of first-strand cDNA and immunoblotting of protein in embryo extract.

**Figure 2:**
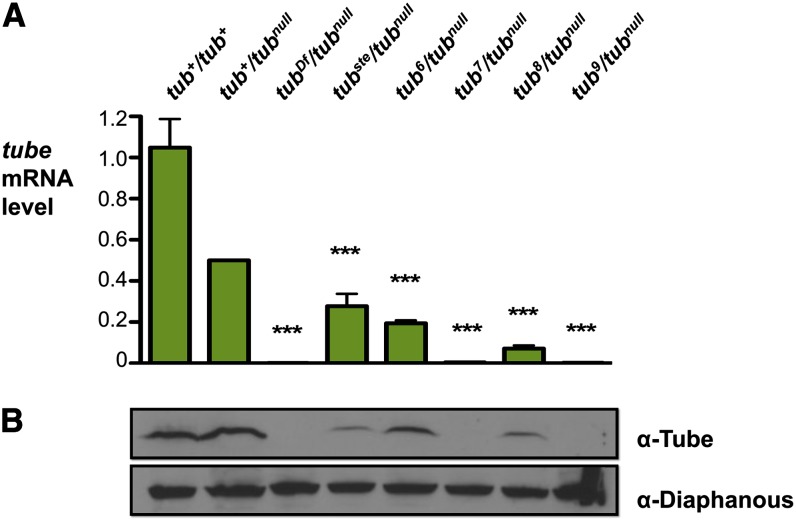
Maternal *tube* expression is dramatically reduced in *tub^var^/tub^null^* females. (A) Quantitation of *tube* mRNA in embryos. Quantitative RT-PCR data of *tube* expression in embryos from females of the specified genotype. Expression data were normalized to *rp49* expression and are presented as a fraction of *tub^+^/tub^null^* expression, with *tub^+^/tub^null^* set to 0.5. Error bars represent S.E.M., ***P < 0.001. (B) Quantitation of Tube protein in embryos. Immunoblot using α-Tube sera of protein isolated from embryos from females of specified genotype. Loading control is α-Diaphanous.

All of the *tub^var^* alleles exhibited a marked mean reduction in *tube* expression. In embryos from *tub^ste^/tub^null^* females, the level of *tube* mRNA was reduced on average to 28% of the wild-type level (Figure 2A). For *tub^6^/tub^null^*, *tub^7^/tub^null^*, *tub^8^/tub^null^*, and *tub^9^/tub^null^*, which exhibit a more severe reduction in *tube* function (see Figure 1), *tube* mRNA levels were only 0.2–19% of the wild-type level (Figure 2A). Immunoblotting revealed that Tube protein levels also were greatly reduced (Figure 2B). Furthermore, Tube protein levels correlated closely with mRNA levels. On the basis of these findings, we concluded that the mutations responsible for the *tub^var^* phenotypes reduce the production of *tube* mRNA.

The finding that *tube* expression is strongly reduced in *tub^var^* mutants is consistent with a model in which phenotypic variability originates from normally occurring, probabilistic fluctuations in transcription. It remained a possibility, however, that the mutations increase transcriptional noise in addition to reducing average transcription levels. To distinguish between these two models, we measured *tube* expression in individual embryos from *tub^ste^/tub^null^*, *tub^8^/tub^null^*, and control females by qRT-PCR.

As shown in Table 1, the *tub^var^* alleles do not enhance transcriptional fluctuations of *tube*. Rather, the standard deviations (SDs) of *tube* expression from the *tub^var^* chromosomes were comparable to each other and to the wild type. This finding demonstrates that the mutations responsible for the *tub^var^* phenotypes reduce *tube* expression without introducing additional variability in gene expression. Said another way, the phenotype of embryos from *tub^var^* females unmasks transcriptional noise.

**Table 1:** Maternal *tube* expression is equally variable in *tub^var^* and wild-type females

Maternal Genotype	Phenotype	Mean (×10^−4^)	SD (×10^−4^)
*tub^+^/tub^null^*	Wild-type	25	11
*tub^ste^/tub^null^*	Variable	23	9.4
*tub^8^/tub^null^*	Variable	8.4	8.6
*tub^4^/tub^null^*	Strongly dorsalized	3.1	1.4

Quantitation of *tube* mRNA in individual embryos from females of the specified genotype by qRT-PCR. Expression data were normalized to *rp49* expression. n ≥ 32 for each genotype. qRT-PCR, quantitative real-time polymerase chain reaction.

We also included in our analysis the allele *tub^4^*, which produces reduced levels of functioning Tube protein (Letsou *et al.* 1993) and yet displays an invariant dorsalization phenotype (see Figure 1C). The *tub^4^* chromosome contains a mutation that disrupts splicing, leading to only a very small fraction of *tube* transcripts being properly spliced and consequently very low amounts of functioning Tube protein. This finding would suggest that low *tube* gene product levels are required but not sufficient for a variable phenotype. Upon analyzing RNA from individual embryos produced by *tub^4^/tub^null^* females, we found that the average level of spliced *tube* mRNA for *tub^4^* was greatly reduced relative to the wild type (Table 1). Furthermore, we found that the SD of *tube* expression was considerably less than observed from the other genotypes (Table 1). In other words, spliced *tube* transcript levels varied less from embryo to embryo for *tub^4^* than for other genotypes.

On the basis of our sequence analysis of the transcribed region, it is likely that the *tub^var^* chromosomes are altered for *tube* transcription initiation or elongation, rather than a cotranscriptional or post-transcriptional process. In contrast, the *tub^4^* chromosome displays defective splicing, a cotranscriptional process, but little variation in mature transcript level. This result can best be explained if the *tub^4^* splicing defect acts as a bottleneck, masking fluctuations in transcription initiation.

We drew two conclusions from the comparison of *tub^var^* and *tub^4^* expression in individual embryos. First, because the *tub^4^* chromosome displayed a reduced average *tube* expression level with a lower standard deviation than observed from the other genotypes, qRT-PCR of individual embryos introduced little, if any, additional technical variability. Second, low transcript levels do not inherently cause phenotypic variability. Rather, reduced transcript levels combined with naturally occurring transcriptional fluctuations generate the *tub^var^* phenotypic variability.

### Retrotransposon-mediated disruption of *tube* expression

**Figure 3:**
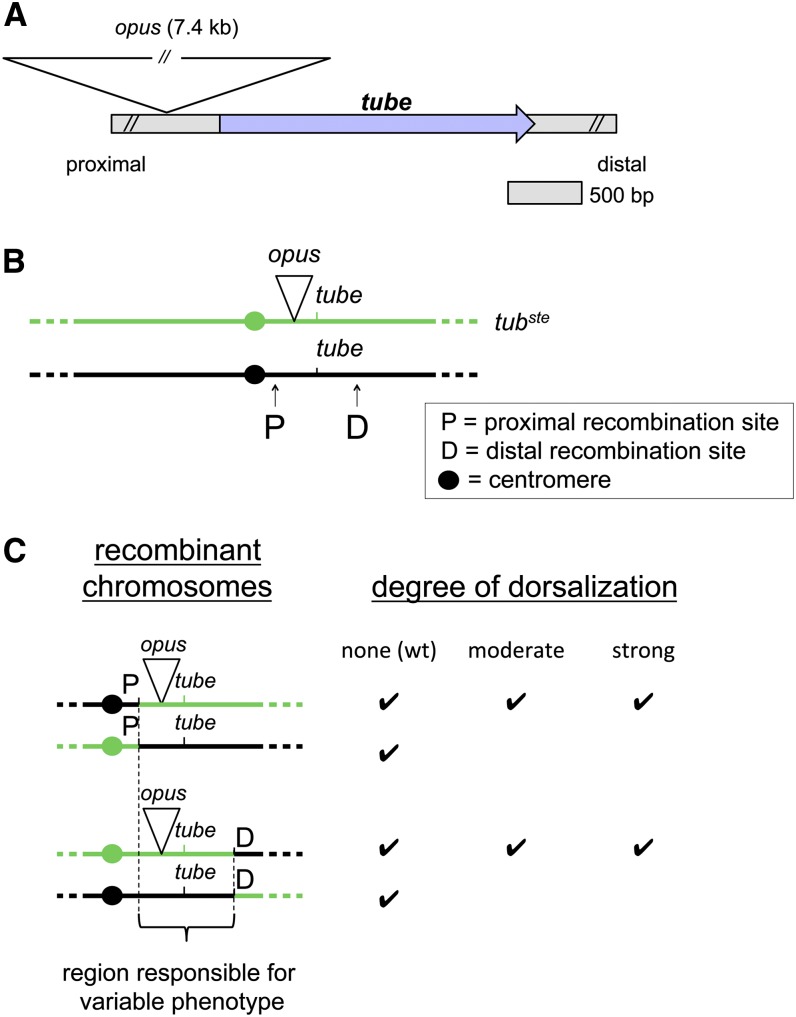
An *opus* retrotransposon insertion 301 bp upstream of *tube* causes variable dorsalization. (A) Schematic of *opus* insertion site relative to the *tube* gene in the *tub^var^* chromosomes. (B) Schematic of site-specific recombination technique used to map the mutation responsible for the variable dorsalization in the *tub^ste^* chromosome. Schematic is not to scale. (C) Phenotypic data from site-specific recombination mapping of the *tub^ste^* chromosome. Recombination was induced at a *P*-element insertion site located either 8 kb proximal (P) or 8 kb distal (D) to the *tube* gene. Phenotypes were assayed in embryos from females bearing the recombinant chromosome in *trans* to *tub^null^*.

Our investigation revealed that maternal *tube* expression was diminished in all five *tub^var^* alleles, suggesting that the phenotypic variability is caused by mutations that reduce *tube* transcription. We therefore set out to find *cis*-regulatory mutations affecting *tube*. We have previously demonstrated that a transgene that includes the *tube* transcription unit and 8 kb of DNA directly upstream rescues Toll signaling in *tube* deficient embryos (Letsou *et al.* 1991). We began by amplifying this region of the *tub^ste^* chromosome with conventional and TAIL PCR (see *Material and Methods* and Rakyan and Beck 2006), followed by sequencing. In this manner, we discovered a retrotransposon insertion at position −301 relative to the *tube* transcription start site (Figure 3A). By PCR-based analysis, we further found that each of the *tub^var^* chromosomes, including *tub^6^*, contains this −301 insertion but wild-type chromosomes do not (Figure S3). The retrotransposon is a member of a family of insertions called *opus* elements, which are LTR-containing retrotransposons typically found in 20-30 copies distributed throughout the *D. melanogaster* genome (Kaminker *et al.* 2002).

Given the finding that all of the *tub^var^* alleles contain the *opus* insertion, we made two predictions. First, because the *opus* insertion is approximately 7.4 kb and is located in the *tube* promoter region, it is likely required for the variable phenotype observed in all *tub^var^* alleles. In this case, the variable phenotype should map to a region containing the *opus* insertion. Second, because each allele displays a unique phenotypic profile, the *opus* insertion is probably the primary event but cannot be the sole source of the variable phenotype. Instead, we hypothesized that the *tub^var^* chromosomes, perhaps with the exception of *tub^ste^*, are doubly mutant for *tube*. In this case, we should be able to identify additional, enhancing mutations in some or all of the *tub^var^* chromosomes that are required for their unique phenotypic profiles.

To address the first prediction—whether the *opus* insertion is required for the *tub^var^* phenotypes—we performed mapping by site-specific male recombination (Chen *et al.* 1998). We hypothesized that the *tub^ste^* chromosome might not contain a second mutation because it is the least affected of the *tub^var^* alleles with regard to *tube* expression and embryonic phenotype. We therefore began by mapping the mutation responsible for the *tub^ste^* phenotype. Specifically, we induced recombination between the *tub^ste^* chromosome and a chromosome bearing either of two *P* element insertions, one approximately 8 kb upstream and one approximately 8 kb downstream of the *tube* transcription unit (Figure 3B). As shown in Figure 3C, these studies demonstrated that a 25-kb region encompassing *tube* and the *opus* insertion was both necessary and sufficient to generate the range of dorsalized phenotypes associated with *tub^ste^*. Furthermore, sequencing of the entire 25-kb region in the *tub^ste^* chromosome revealed just seven other changes, each of which were minor sequence variations when compared to wild-type cDNA or genomic sequences (Table S1). These findings are consistent with the hypothesis that the *opus* retrotransposon is the primary event responsible for the *tub^var^* phenotypic variability and acts by diminishing *tube* expression.

Using qRT-PCR and 5′ RACE in combination with published modENCODE RNAseq data we determined that the *opus* insertion disrupts *tube* expression without affecting the position of the transcription start site (Figure S4). Moreover, the *opus* insertion site separates the *tube* transcription start site from the most highly conserved intergenic region upstream of *tube* (Celniker *et al.* 2002; Kent *et al.* 2002). It thus appears that the *opus* insertion disrupts *tube* regulation, reducing *tube* expression without compromising the boundaries of the *tube* transcript.

### Context-specific expression defects

**Figure 4:**
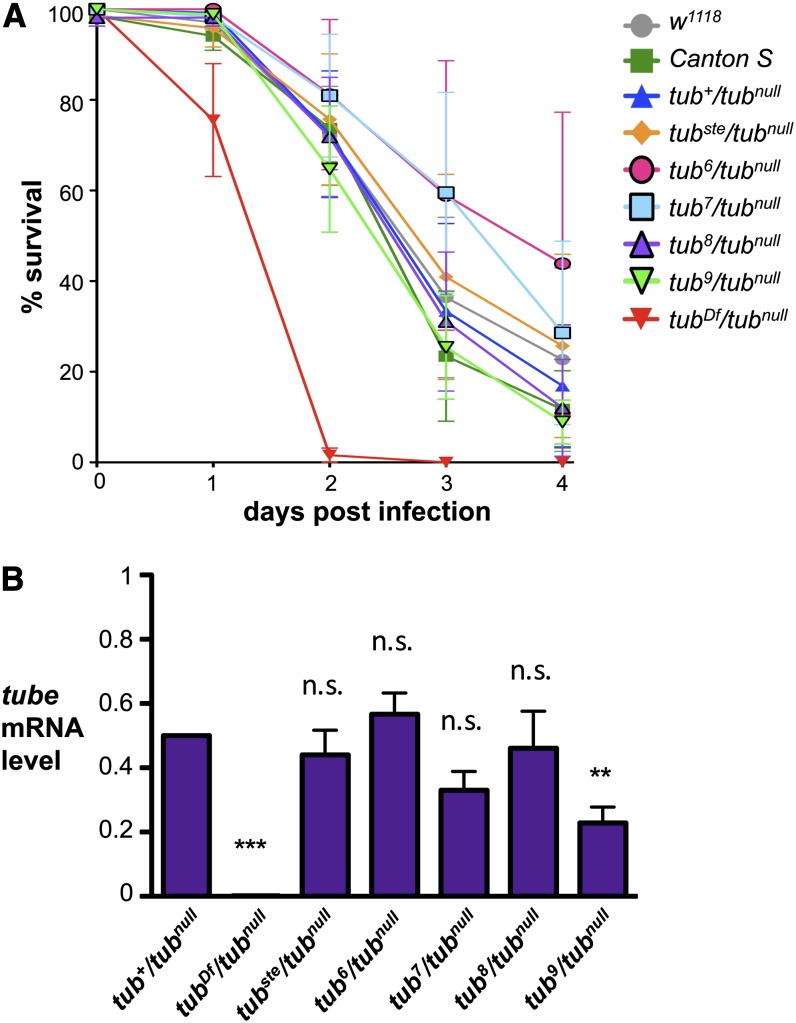
Immune function of *tube* appears unaffected in *tub^var^* adults. (A) Survival of adult males of specified genotype after septic wounding with *F. oxysporum* spores. Analysis of *tub^7^* was performed in *trans* to *tub^2^*, a strong hypomorphic allele. Error bars represent SEM. (B) Quantitation of *tube* mRNA in adults. Quantitative RT-PCR data of *tube* expression in adult males of the specified genotype. Expression data were normalized to *rp49* expression and are presented as a fraction of *tub^+^/tub^null^* expression, with *tub^+^/tub^null^* set to 0.5. Error bars represent SEM. **P < 0.01, ***P < 0.001, n.s. = not significant.

The aforementioned findings indicate that the presence of the *opus* insertion alters *tube* expression. Because Tube is required for Toll pathway function in both embryonic patterning and innate immunity, we wondered whether *tub^var^* mutants also display immune defects. To answer this question, we assayed *tub^var^/tub^null^*, *tub^null^/tub^null^*, and wild-type adult males for survival after septic injury with the fungus *Fusarium oxysporum*, a specific inducer of Toll signaling. We found that although *tub^null^/tub^null^* males died within 2 d after infection, *tub^var^/tub^null^* males survived on average for 4 d following infection, indistinguishable from wild-type males (Figure 4A). Thus, the *tub^var^* chromosomes detectably disrupt Toll signaling in embryos but not in adult immune tissues.

We considered two explanations for the distinct effects of the *tub^var^* chromosomes in different *in vivo* contexts. One possibility is that the threshold level of Tube required for full pathway function during infection is lower than during embryonic development, allowing *tub^var^/tub^null^* flies to mount an effective immune response with relatively small amounts of Tube. Alternatively, the effect of the *opus* insertion on *tube* expression could differ among tissues. To distinguish between these hypotheses, we measured *tube* mRNA levels by qRT-PCR in whole adult males. For four of the five variable alleles, *tub^var^/tub^null^* adult males exhibited wild-type levels of *tube* expression (Figure 4B). In the case of *tub^9^*, expression in adult males was somewhat reduced compared to *tub^+^/tub^null^*, but far greater than that observed in embryos. In all cases, it seems that the *opus* insertion affects the ability of cells to transcribe *tube* in the ovary but not in the immune tissues. Expression of *tube* in the different tissues presumably requires distinct regulatory elements, making the effect of the *opus* element on *tube* expression context-specific.

### Additional mutations and enhancement of the variable phenotype

As stated previously, we postulated that the synergistic interactions of the *opus* insertion with an additional mutation on each of the *tub^var^* chromosomes (except for *tub^ste^*) cause the distinct phenotypic profile of each allele. As described above, the *tub^6^* chromosome carries a missense mutation in addition to the *opus* insertion. We were unable to identify a second mutation in the regions flanking *tube* in the *tub^8^* chromosome (data not shown). However, we were successful in identifying an additional and likely significant mutation in both the *tub^7^* and *tub^9^* chromosomes, both of which had strikingly reduced levels of *tube* mRNA (see Figure 2).

**Figure 5:**
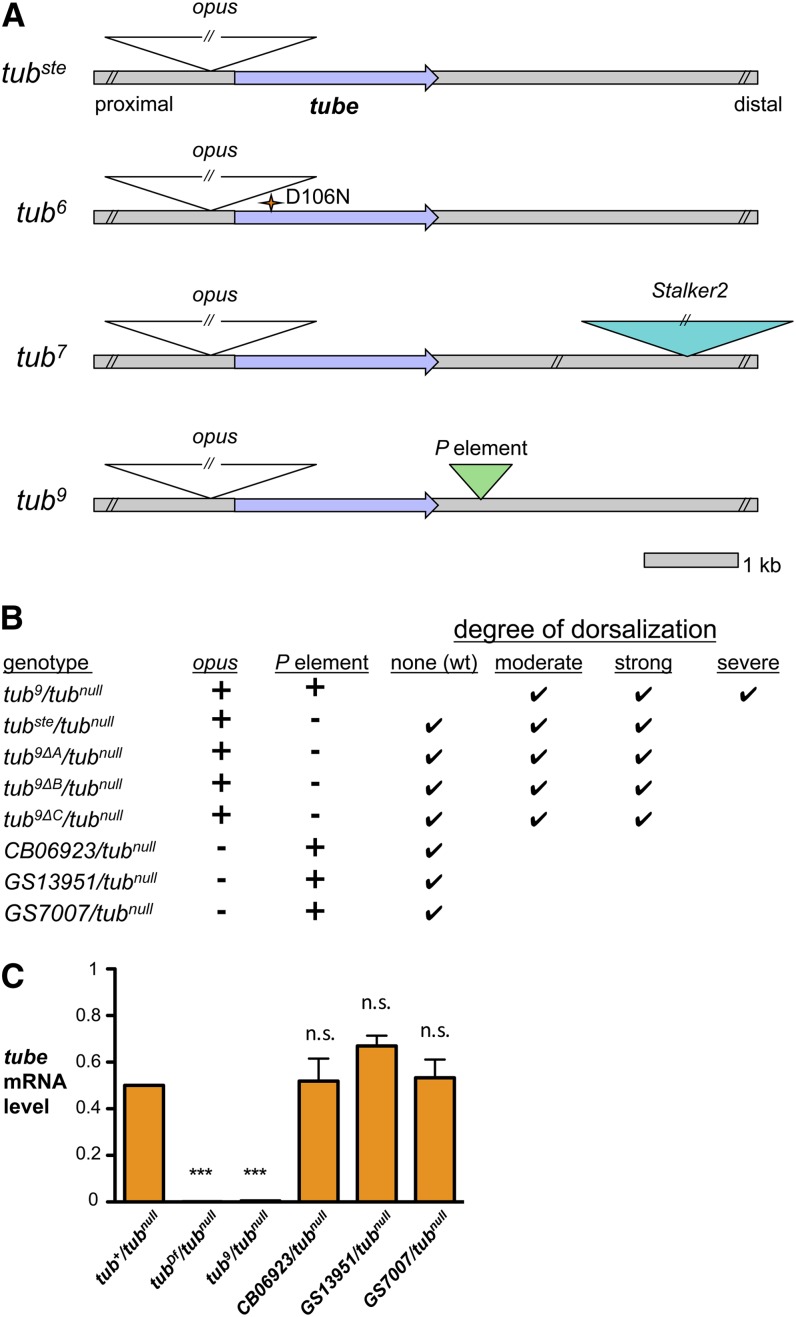
Additional mutations in the *tub^var^* chromosomes genetically interact with the *opus* insertion to enhance the variable phenotype. (A) Schematic of locations of identified mutations in *tub^var^* chromosomes, including *opus* insertion, *tub^6^* missense mutation, *tub^7^ Stalker2* insertion, and *tub^9^ P*-element insertion. (B) Phenotypic ranges of embryos from females of specified genotype in *trans* to *tub^null^*. Precise excisants (*tub^9Δ^*) were generated using a transposase source (Δ2-3). *GS7007*, *GS13951*, and *CB06923* are chromosomes containing *P*-element insertions within 4 bp of the *tub^9^ P*-element insertion site. (C) Quantitation of *tube* mRNA in embryos from females bearing *P* elements downstream of *tube*. Quantitative RT-PCR expression data were first normalized to *rp49* expression and are presented as a fraction of *tub^+^/tub^null^* expression, with *tub^+^/tub^null^* set to 0.5. Error bars represent SEM. ***P < 0.001, n.s. = not significant.

For the *tub^7^* chromosome, we analyzed the 25-kb region indicated by site-specific recombination experiments to be responsible for the variable phenotype on the *tub^ste^* chromosome. Using an approach combining conventional PCR, TAIL-PCR, and sequencing, we identified an insertion of *Stalker2*, another retrotransposon, 6 kb downstream of the *tube* transcription unit in the *tub^7^* chromosome (see Figure 5A). *Stalker2* is an LTR-containing retrotransposon found in approximately 10 copies distributed over the *D. melanogaster* genome (Kaminker *et al.* 2002). Genomic sequencing of this 25-kb region revealed no differences between *tub^7^* and *tub^ste^* other than the *Stalker2* insertion. Furthermore, PCR-based analysis demonstrated that the *Stalker2* insertion is absent from all other *tub^var^* chromosomes and from wild-type chromosomes (Figure S5). Thus, the *Stalker2* insertion appears to be the second mutation in the *tub^7^* chromosome that interacts with the *opus* insertion to generate its unique phenotype.

In the case of *tub^9^*, Hecht and Anderson had found a *P*-element insertion downstream of the *tube* gene (Hecht and Anderson 1993). By sequencing we confirmed the presence of a *P* element, 687 bp long, located 581 bp downstream of the end of the *tube* transcription unit (see Figure 5A). To test whether this *P* element synergistically interacts with the *opus* insertion, we mobilized the *P* element and assayed for precise excision. We obtained three such excisants and, as reported by Hecht and Anderson, they were fertile (Hecht and Anderson 1993). However, we found that the phenotypic distribution of embryos from the excisant females was not wild-type, as previously reported, but was instead variable and similar to that of embryos from *tub^ste^*/*tub^null^* females (Figure 5B). Thus, the excision of the *P* element partially restored *tube* function in these embryos. This finding demonstrates that the unique *tub^9^* phenotypic profile reflects the combined activities of two mutations, the *opus* and *P* element insertions. The *opus* element is the primary event, and the *P* element additionally decreases *tube* expression and correspondingly enhances the dorsalization phenotype of *tub^9^* relative to *tub^ste^*.

Given the proximity of the *P*-element insertion in *tub^9^* to the tube gene, we wondered whether such an insertion by itself would perturb *tube* expression. We could not, however, remove the *opus* insertion from *tub^9^* because retrotransposons do not excise. Instead, we analyzed *P*-element insertions in the same location as the *P* element in *tub^9^* but in a background devoid of the *opus* insertion. Taking advantage of available collections, we obtained three such *P*-element insertions, each within four base pairs or less of the *tub^9^ P*-element insertion site. All conferred wild-type *tube* function during embryonic patterning (Figure 5B). Furthermore, *tube* mRNA levels were wild-type in embryos from females bearing these *P* elements (Figure 5C). Hence, a *P*-element insertion at the same location as that in *tub^9^* does not by itself have an effect on *tube* expression. The simplest explanation for these results is that the *P* element in *tub^9^* exerts its effect on *tube* expression exclusively through its synergistic interaction with the upstream *opus* insertion.

## Discussion

### Sensitizing mutations and synergistic effects of additional mutations

We have identified a retrotransposon insertion upstream of the *tube* transcription start site that is specific to the *tub^var^* alleles and that lies within the region responsible for the variable phenotype of *tub^ste^*. We conclude that the *opus* insertion at −301 dramatically reduces *tube* expression, producing a variable phenotype that depends on naturally fluctuating transcriptional levels. The *opus* insertion is approximately 7.4-kb long and sits between the *tube* transcription start site and the region of upstream intergenic sequence with the highest conservation among Drosophila species (Celniker *et al.* 2002; Kent *et al.* 2002). This conserved region is most likely a regulatory element, suggesting a means by which the *opus* insertion could interrupt *tube* expression. The *opus* insertion could act by simply spatially separating the *tube* transcription start site from an important regulatory element. Alternatively, the *opus* insertion could induce epigenetic changes that inhibit the interaction of this conserved region with DNA binding proteins that promote *tube* transcription.

Given that all of the *tub^var^* alleles contain the *opus* insertion and several other small polymorphisms absent from the reference genome, it seems likely that a common progenitor chromosome was used to generate each of the *tub^var^* alleles (Hecht and Anderson 1993). We speculate that the *opus* insertion provided a sensitized background for mutagenesis, leading to the recovery of the *tub^6^*, *tub^7^*, *tub^8^*, and *tub^9^* chromosomes. Our data support a model in which each of the *tub^var^* chromosomes, except *tub^ste^*, contain a second, enhancing mutation, which acts synergistically with the *opus* insertion to substantially disrupt *tube* gene function. Despite the fact that *tub^8^* also displays a phenotype distinct from that observed in *tub^ste^*, we have not identified a second mutation in the *tub^8^* chromosome. It may be, therefore, that the *tub^8^* chromosome contains multiple or complex changes that cannot be as easily dissected by site-specific recombination mapping.

We identified two different types of mutations that interact with the *opus* insertion to increase the severity of the variable dorsalization. The second mutation on the *tub^6^* chromosome, a missense mutation, appears to mildly decrease Tube protein function without affecting *tube* expression. Embryos from *tub^6^/tub^null^* females are more severely dorsalized, on average, than those from *tub^ste^/tub^null^* females despite similar mRNA levels (see Figure 2A). Embryos from *tub^6^/tub^null^* females are not completely dorsalized, however, demonstrating that the Tube protein encoded by *tub^6^* retains at least some activity. The wild-type survival of *tub^6^/tub^null^* males following fungal infection further demonstrates this functionality. We speculate that their wild-type survival is due to the elevated levels of *tube* gene product in males relative to embryos. An excess of Tube protein in these males would mitigate the shortcoming of reduced Tube activity. In the embryos, however, low Tube protein levels in combination with reduced functionality generate the unique phenotypic profile of *tub^6^*.

In the cases of *tub^7^* and *tub^9^*, we identified a second transposable element insertion in each chromosome, which we believe work synergistically with the *opus* insertion to further reduce *tube* mRNA levels. The insertions that we found in the course of our studies of the *tub^var^* chromosomes represent three distinct families of transposable elements, giving us examples of the complexity of genetic interactions that are possible among transposable elements. Each element contains distinct sets of *cis* regulatory sequences, which on a genome-wide scale, allows for seemingly endless combinations of potential genetic perturbations of a gene locus via alterations to the local chromatin landscape.

Our studies of the *tub^9^* chromosome provide evidence that transposable element insertions that are innocuous on their own can induce profound alterations in local gene expression when located near other transposable element insertions. If a single insertion is benign on its own, such as the *tub^9^ P* element, it will not be selected against. This safety from selection allows transposable element insertions to accumulate around the genome, sensitizing many loci to additional insertions. One might expect more published examples of similar hypervariable phenotypes given the abundance of transposable elements in eukaryotic genomes. We speculate, however, that in-depth studies of such mutations are underrepresented in the literature because they lack the robust phenotypes required by most traditional genetic approaches.

### Gene-proximal transposable elements and gene regulation

The fact that gene-proximal transposable element insertions can cause dramatic and complex regulatory changes on neighboring genes is relevant to our understanding of intergenic DNA. Transposable elements comprise approximately 10–20% of the *D. melanogaster* genome and more than 45% of the human genome (Burns and Boeke 2012; Ganko *et al.* 2006). The *D. melanogaster* genome contains at least 96 families of transposable elements, each identified based on their unique sequence composition and each ranging in euchromatic copy number from 1 to approximately 150 per genome (Kaminker *et al.* 2002). In wild populations, the frequency with which transposable element insertion sites are shared is low (Biemont *et al.* 1994; Charlesworth and Lapid 1989; Charlesworth *et al.* 1992) or, in other words, subpopulations of flies harbor unique collections of transposable element insertion sites. The diversity of insertion sites produces many opportunities for adaptive modulations in gene expression.

In the case of the *tub^var^* alleles, the transposable element insertions produce a detrimental phenotype. However, a similar effect on a non-essential gene could lead to a tempered modulation of gene activity, a change that could potentially improve organismal fitness. Such effects have been reported in both *D. melanogaster* and mammalian models involving particular subsets of neurons that show elevated transposition rates (Perrat *et al.* 2013; Thomas and Muotri 2012). In at least one case, the resulting *de novo* insertions altered local gene expression and cell fate (Muotri *et al.* 2005). One possibility is that derepression of transposon mobility is an evolutionary adaptation to generate genomic diversity, and subsequently gene expression diversity, among genetically identical cells.

### Surplus gene expression as a source of phenotypic robustness

We find that *tub^ste^/tub^null^* females on average express only 28% of the wild-type level of maternal *tube* transcripts and yet approximately 82% of their embryos develop wild-type dorsoventral axes. This finding suggests that the threshold of *tube* expression needed for a wild-type phenotype is considerably less than 50% of the wild-type level. It seems energetically unfavorable for an organism to produce so much more mRNA and protein than necessary. However, surplus gene expression may be a molecular mechanism to buffer the effects of stochastic influences. This model is simpler than the alternative of attempting to minimize or eliminate fluctuations in transcription factor abundance or activity.

In the case of early embryonic development, the guaranteed abundance of a gene product above a threshold level is particularly important because there is no opportunity for feedback regulation—all germline gene expression is completed before fertilization. Thus, a mutation that significantly perturbs maternal gene expression would have an irreversible effect on gene product levels in the oocyte and, ultimately, the phenotype of the progeny.

It would be interesting to look for the extent of surplus expression in other maternally expressed genes, especially those essential to embryonic survival, like *tube*, and compare them to the expression profiles of zygotically expressed genes. One possibility is that surplus expression is less common among zygotically expressed genes because there is the opportunity for positive feedback regulation. In this case, the female germline would have unique epigenetic or transcriptional mechanisms to ensure surplus expression of gene products that are to be transferred into the oocyte. A more complete understanding of this problem will require the determination of threshold levels of gene expression for additional genes.

## Supplementary Material

Supporting Information
