# Supplementary material for: Combinatorial Effects of Transposable Elements on Gene Expression and Phenotypic Robustness in Drosophila melanogaster Development
Source: G3 (Bethesda). 2013 Sep 1;3(9):1531–8. doi: 10.1534/g3.113.006791 (PMC3755913; doi:10.1534/g3.113.006791)
Supplement: Supporting Information [file supp_3_9_1531__index.html]

Combinatorial Effects of Transposable Elements on Gene Expression and Phenotypic Robustness in Drosophila melanogaster Development — Supporting Information 

# Combinatorial Effects of Transposable Elements on Gene Expression and Phenotypic Robustness in *Drosophila melanogaster* Development

## Supporting Information for Clemmons and Wasserman, 2013

**Files in this Data Supplement:**

- Supporting Information - Figures S1-S5 and Table S1 (PDF, 11.5 MB)
- Figure S1 - The spectrum of embryonic dorsalization phenotypes (PDF, 7.2 MB)
- Figure S2 - Embryos from single females display a range of phenotypes, as is also seen among embryos produced from a population of females of the same genotype (PDF, 718 KB)
- Figure S3 - The *opus* insertion is present in all *tubvar* chromosomes and absent from wild-type chromosomes (PDF, 233 KB)
- Figure S4 - Downregulation of *tube* by *opus* insertion does not depend on an alternative transcription start site (PDF, 807 KB)
- Figure S5 - The downstream *Stalker2* insertion appears to be unique to *tub7* (PDF, 2.7 MB)
- Table S1 - Polymorphisms found in *tubste* chromosome within the 25 kb region determined via site-specific recombination to be responsible for the *tubste* variable phenotype (PDF, 67 KB)
