# Supplementary material for: Combinatorial Effects of Transposable Elements on Gene Expression and Phenotypic Robustness in Drosophila melanogaster Development
Source: G3 (Bethesda). 2013 Sep 1;3(9):1531–8. doi: 10.1534/g3.113.006791 (PMC3755913; doi:10.1534/g3.113.006791)
Supplement: Supporting Information [file supp_g3.113.006791_006791SI.pdf]

G3 Genes | Genomes | Genetics

Combinatorial effects of transposable elements on gene expression and phenotypic robustness in *Drosophila melanogaster* development.

Alexa W. Clemmons and Steven A. Wasserman

Section of Cell & Developmental Biology, University of California San Diego La Jolla, California 92093---0349

DOI: 10.1534/g3.113.006791

Table S1. Polymorphisms found in tub^{ste} chromosome within the 25 kb region determined via site-specific recombination to be responsible for the tub^{ste} variable phenotype. Sequence polymorphisms are relative to the reference genome and a functional *tube* cDNA (Celniker *et al.* 2002; Letsou *et al.* 1993); all were also found in tub^{7} and tub^{8}. Locations of site-specific recombination sites and of the beginning and end of the *tube* transcription unit are noted under Reference Points.

Polymorphisms

**Description**	**Location**
4 bp deletion (TTAG)	3R: 205,032 - 205,035
Single base substitution (A > G)	3R: 206,259
1 bp deletion (T)	3R: 207,180
4 bp deletion (ATTT)	3R: 213,067 - 213,070
7,400 bp insertion (*opus*)	3R: 213,161
Single base substitution (T > A)	3R: 219,883
Single base substitution (G > C)	3R: 220,488
Two base substitution (GC > AA)	3R: 223,222 - 223,223

Reference points

**Description**	**Location**
Proximal recombination site	3R: 204,648
tube transcription start site	3R: 213,460
End of *tube* transcription unit	3R: 215,536
Distal recombination site	3R: 223,611

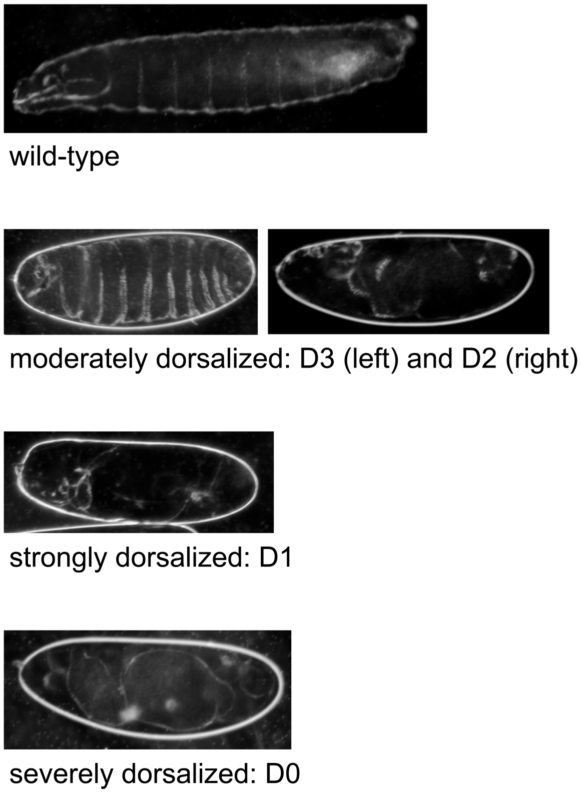

Figure S1. The spectrum of embryonic dorsalization phenotypes.

Pictures of embryos representative of the dorsalization phenotypes referred to in this paper. Embryonic phenotypes were scored based on pre-existing scale, D0-D3 and wild-type (Wieschaus and Nüsslein-Volhard 1986).

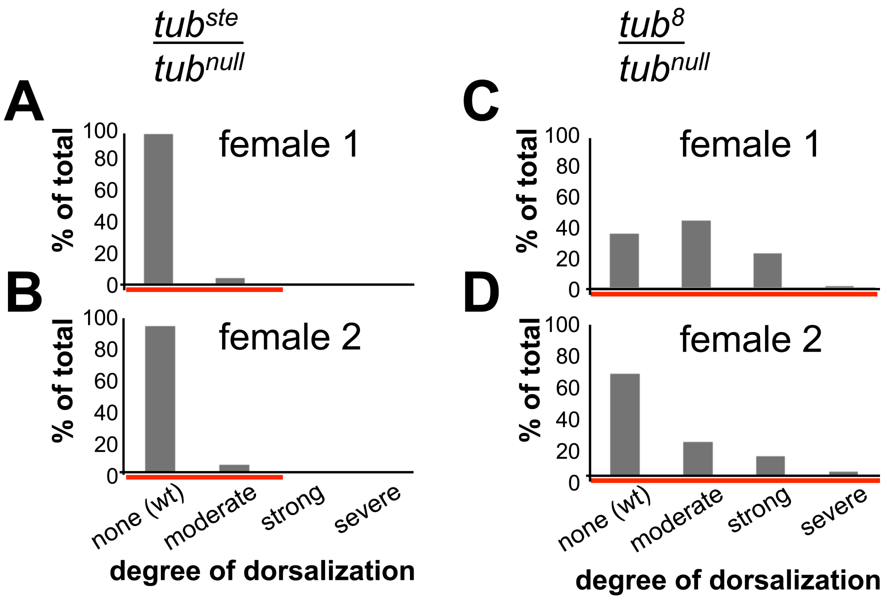

Figure S2. Embryos from single females display a range of phenotypes, as is also seen among embryos produced from a population of females of the same genotype.

Single females of specified genotypes were mated with wild-type males. Cuticle phenotypes are represented as the percent of total embryos scored from that female. Compare to Figure 1. **A.** tub^{ste}/tub^{null} female, n = 156. **B.** tub^{ste}/tub^{null} female, n = 176. **C.** tub^{8}/tub^{null} female, n = 73. **D.** tub^{8}/tub^{null} female, n = 80. Red lines highlight range of phenotypes. Dorsalized phenotypes are underrepresented relative to wild-type in this experiment because of the difficulty in recovering small numbers of unhatched embryos.

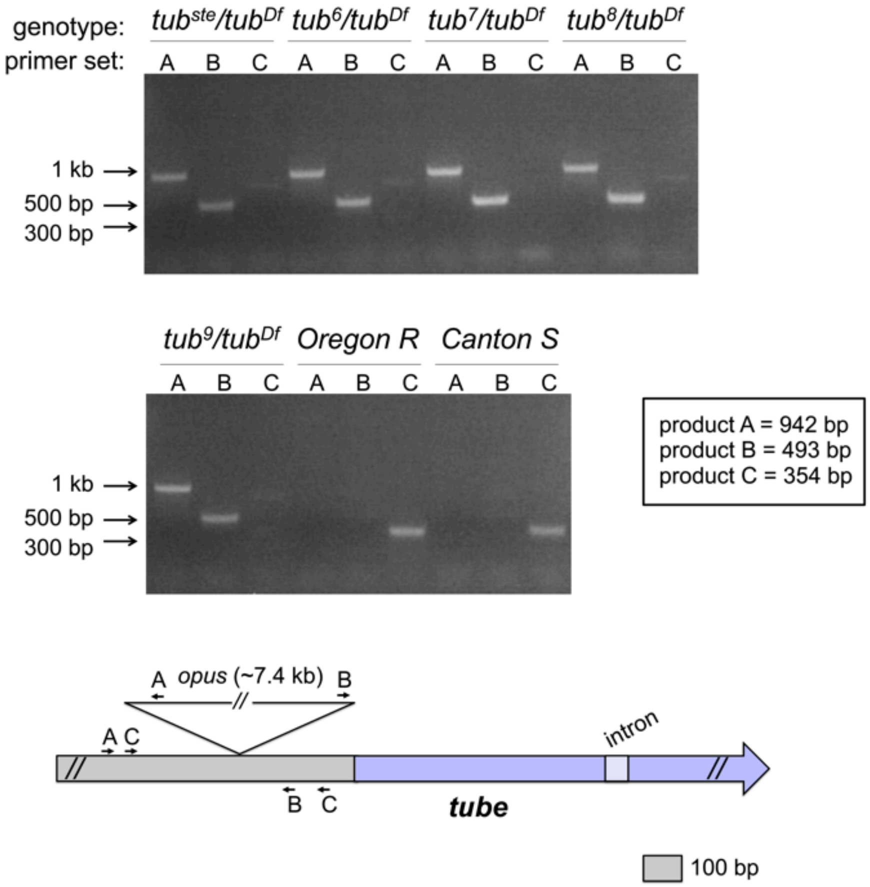

Figure S3. The *opus* insertion is present in all *tub*^{var} chromosomes and absent from wild-type chromosomes. Genomic DNA from flies of the specified genotype was PCR amplified using three different primer sets to assay for the presence of the *opus* insertion (see diagram at bottom). Primer set A spans the 5' insertion junction, primer set B spans the 3' insertion junction, and primer set C spans the entire insertion (>7 kb in *tub*^{ste} chromosome, too large to amplify under given cycling conditions).

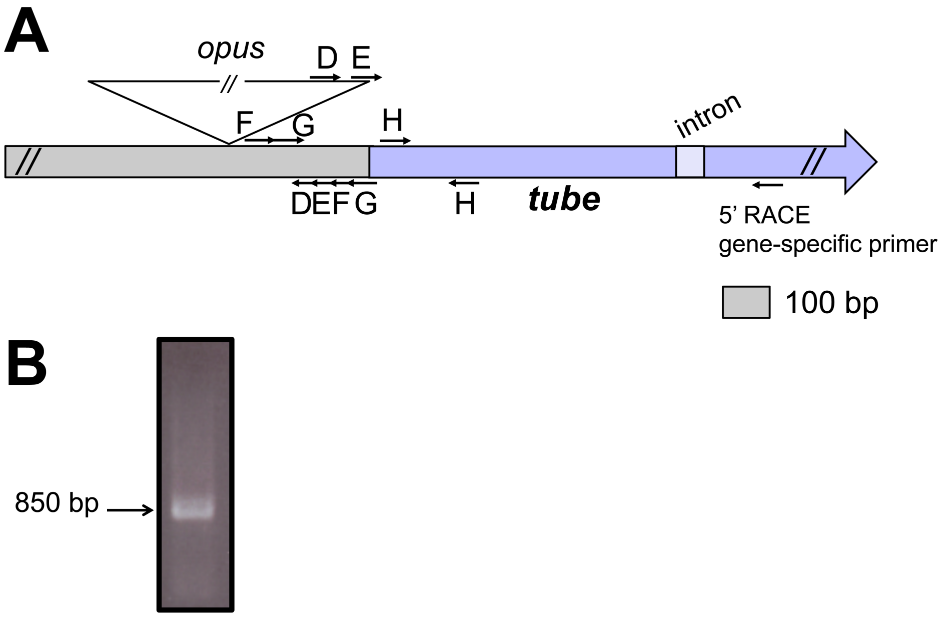

Figure S4. Downregulation of *tube* by *opus* insertion does not depend on an alternative transcription start site.

A. Schematic of primer locations for quantitative RT-PCR and 5’ RACE to assay for use of an alternative transcription start site in embryos from tub^{ste}/tub^{null} females. Quantitative RT-PCR primer sets are labeled D-H. Products were detected from primer set H, but not from primer sets D-G.

B. Results from 5’ RACE using RNA prepared from embryos from tub^{ste}/tub^{null} females. Expected size of *tube* transcript originating from wild-type transcription start size was 830 bp.

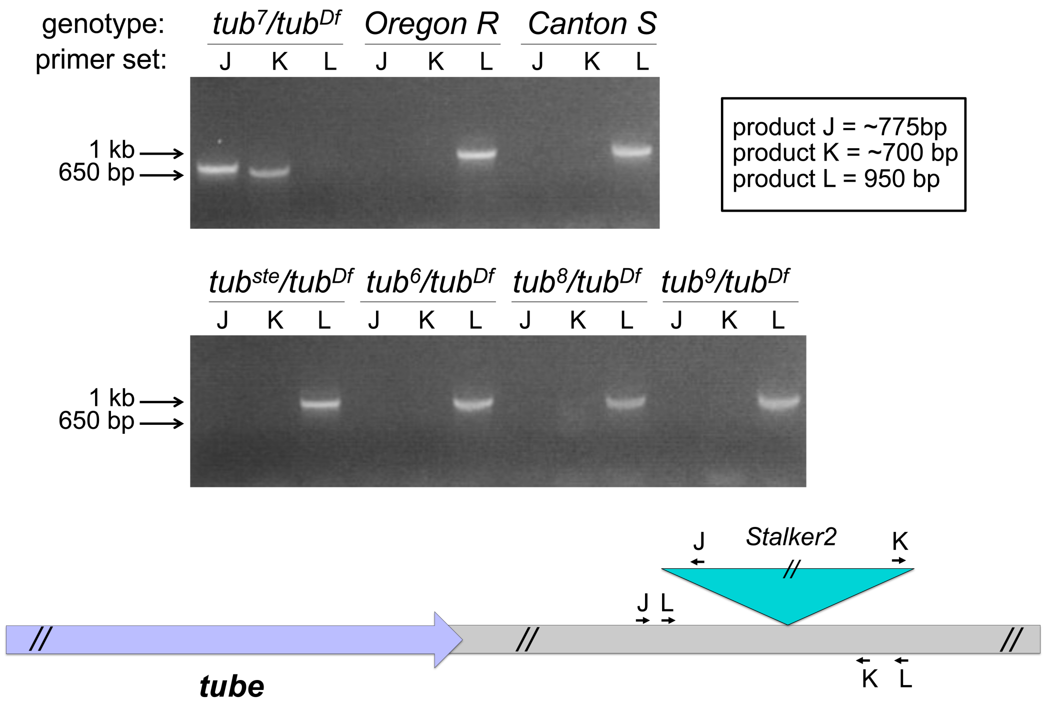

Figure S5. The downstream *Stalker2* insertion appears to be unique to *tub*^{7}.

Genomic DNA from flies of the specified genotypes was PCR amplified using three different primer sets to assay for the presence of the *Stalker2* insertion (see diagram at bottom). Primer set J spans the 5' insertion junction, primer set K spans the 3' insertion junction, and primer set L spans the entire insertion (>8 kb in *tub*^{7} chromosome, too large to amplify using given cycling conditions).
