# Supplementary material for: Combinatorial Effects of Transposable Elements on Gene Expression and Phenotypic Robustness in Drosophila melanogaster Development
Source: G3 (Bethesda). 2013 Sep 1;3(9):1531–8. doi: 10.1534/g3.113.006791 (PMC3755913; doi:10.1534/g3.113.006791)
Supplement: Supporting Information [file supp_g3.113.006791_FigureS1.pdf]

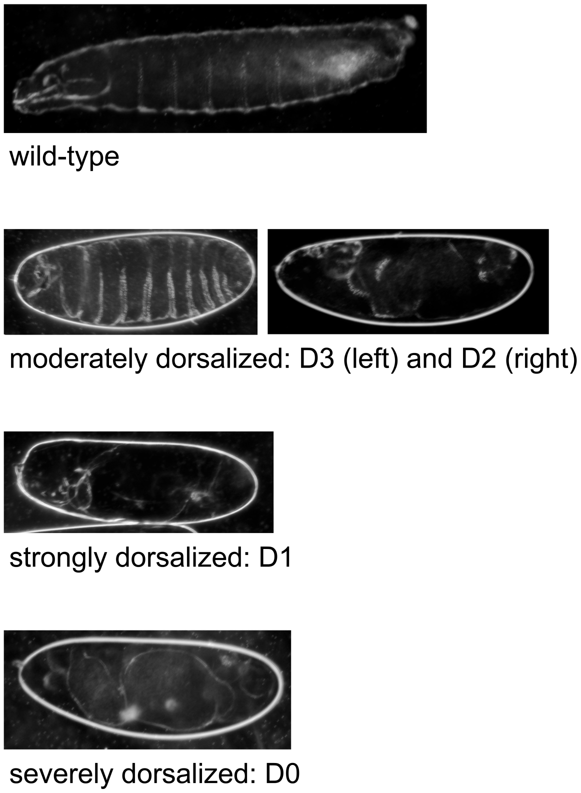

Figure S1. The spectrum of embryonic dorsalization phenotypes.

Pictures of embryos representative of the dorsalization phenotypes referred to in this paper. Embryonic phenotypes were scored based on pre-existing scale, D0-D3 and wild-type (Wieschaus and Nüsslein-Volhard 1986).
