# Supplementary material for: Combinatorial Effects of Transposable Elements on Gene Expression and Phenotypic Robustness in Drosophila melanogaster Development
Source: G3 (Bethesda). 2013 Sep 1;3(9):1531–8. doi: 10.1534/g3.113.006791 (PMC3755913; doi:10.1534/g3.113.006791)
Supplement: Supporting Information [file supp_g3.113.006791_FigureS2.pdf]

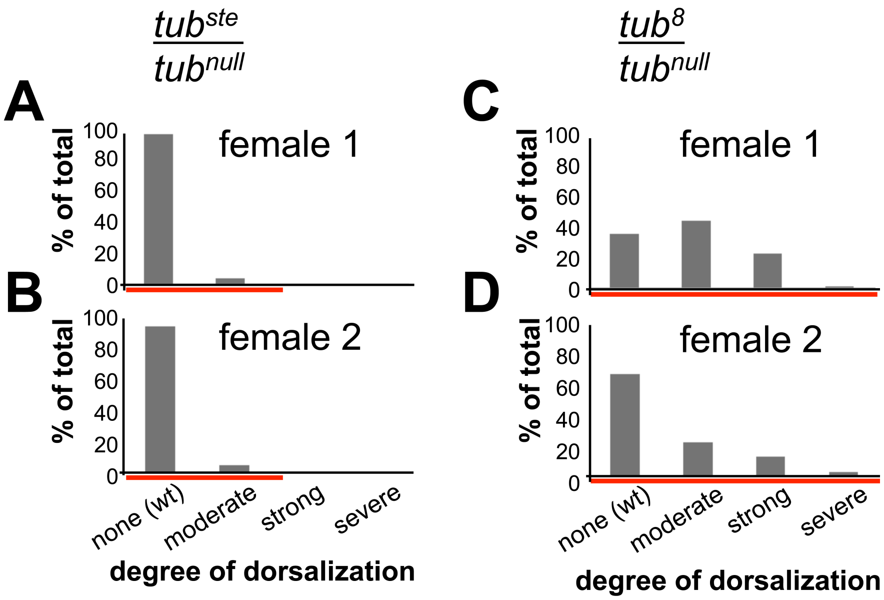

Figure S2. Embryos from single females display a range of phenotypes, as is also seen among embryos produced from a population of females of the same genotype.

Single females of specified genotypes were mated with wild-type males. Cuticle phenotypes are represented as the percent of total embryos scored from that female. Compare to Figure 1. **A.** *tub*^{ste}/*tub*^{null} female, n = 156. **B.** *tub*^{ste}/*tub*^{null} female, n = 176. **C.** *tub*^{8}/*tub*^{null} female, n = 73. **D.** *tub*^{8}/*tub*^{null} female, n = 80. Red lines highlight range of phenotypes. Dorsalized phenotypes are underrepresented relative to wild-type in this experiment because of the difficulty in recovering small numbers of unhatched embryos.
