# Supplementary material for: Combinatorial Effects of Transposable Elements on Gene Expression and Phenotypic Robustness in Drosophila melanogaster Development
Source: G3 (Bethesda). 2013 Sep 1;3(9):1531–8. doi: 10.1534/g3.113.006791 (PMC3755913; doi:10.1534/g3.113.006791)
Supplement: Supporting Information [file supp_g3.113.006791_FigureS3.pdf]

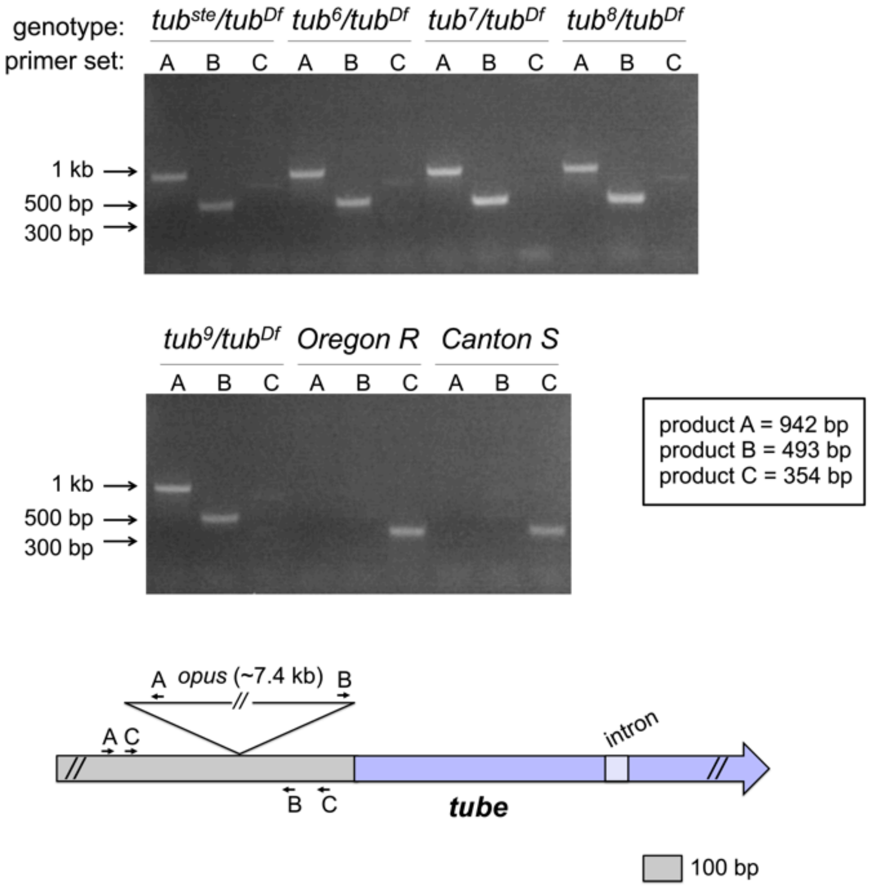

Figure S3. The *opus* insertion is present in all tub^{var} chromosomes and absent from wild-type chromosomes.

Genomic DNA from flies of the specified genotype was PCR amplified using three different primer sets to assay for the presence of the *opus* insertion (see diagram at bottom). Primer set A spans the 5' insertion junction, primer set B spans the 3' insertion junction, and primer set C spans the entire insertion (>7 kb in tub^{ste} chromosome, too large to amplify under given cycling conditions).
