# Supplementary material for: Combinatorial Effects of Transposable Elements on Gene Expression and Phenotypic Robustness in Drosophila melanogaster Development
Source: G3 (Bethesda). 2013 Sep 1;3(9):1531–8. doi: 10.1534/g3.113.006791 (PMC3755913; doi:10.1534/g3.113.006791)
Supplement: Supporting Information [file supp_g3.113.006791_FigureS4.pdf]

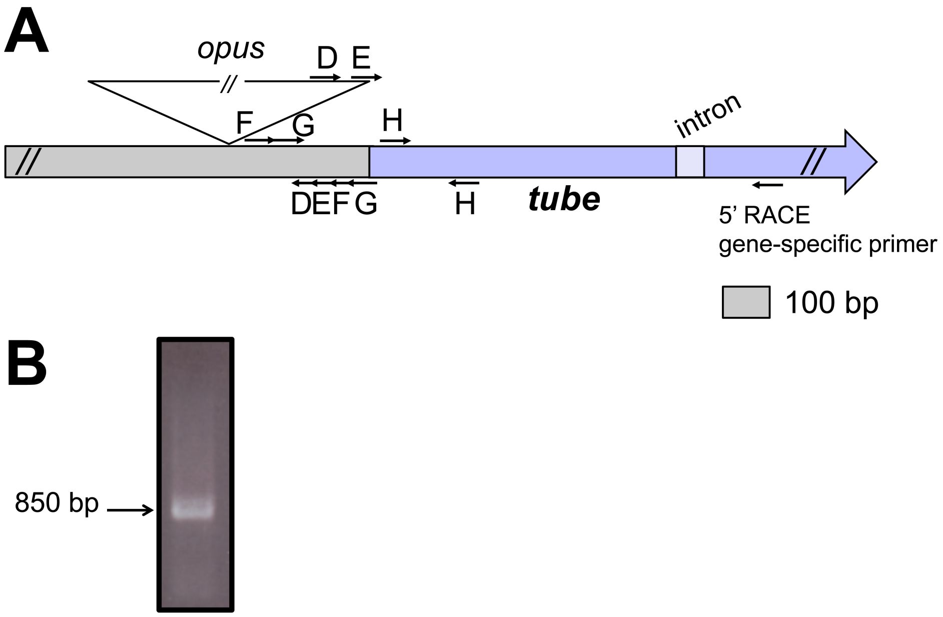

Figure S4. Downregulation of *tube* by *opus* insertion does not depend on an alternative transcription start site.

A. Schematic of primer locations for quantitative RT-PCR and 5' RACE to assay for use of an alternative transcription start site in embryos from tub^{ste}/tub^{null} females. Quantitative RT-PCR primer sets are labeled D-H. Products were detected from primer set H, but not from primer sets D-G.

B. Results from 5' RACE using RNA prepared from embryos from tub^{ste}/tub^{null} females. Expected size of *tube* transcript originating from wild-type transcription start size was 830 bp.
