# Supplementary material for: Combinatorial Effects of Transposable Elements on Gene Expression and Phenotypic Robustness in Drosophila melanogaster Development
Source: G3 (Bethesda). 2013 Sep 1;3(9):1531–8. doi: 10.1534/g3.113.006791 (PMC3755913; doi:10.1534/g3.113.006791)
Supplement: Supporting Information [file supp_g3.113.006791_FigureS5.pdf]

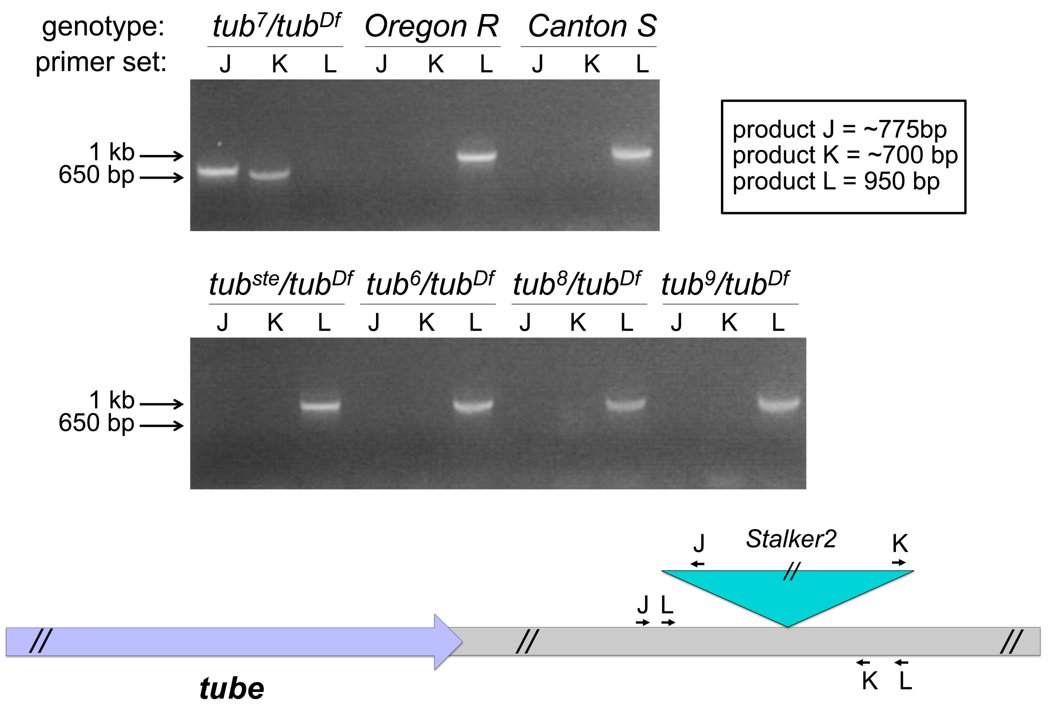

Figure S5. The downstream *Stalker2* insertion appears to be unique to *tub*^{7}.

Genomic DNA from flies of the specified genotypes was PCR amplified using three different primer sets to assay for the presence of the *Stalker2* insertion (see diagram at bottom). Primer set J spans the 5’ insertion junction, primer set K spans the 3’ insertion junction, and primer set L spans the entire insertion (>8 kb in *tub*^{7} chromosome, too large to amplify using given cycling conditions).
