# Supplementary material for: Combinatorial Effects of Transposable Elements on Gene Expression and Phenotypic Robustness in Drosophila melanogaster Development
Source: G3 (Bethesda). 2013 Sep 1;3(9):1531–8. doi: 10.1534/g3.113.006791 (PMC3755913; doi:10.1534/g3.113.006791)
Supplement: Supporting Information [file supp_g3.113.006791_TableS1.pdf]

Table S1. Polymorphisms found in tub^{ste} chromosome within the 25 kb region determined via site-specific recombination to be responsible for the tub^{ste} variable phenotype. Sequence polymorphisms are relative to the reference genome and a functional *tube* cDNA (Celniker *et al.* 2002; Letsou *et al.* 1993); all were also found in tub^{7} and tub^{8}. Locations of site-specific recombination sites and of the beginning and end of the *tube* transcription unit are noted under Reference Points.

Polymorphisms

Description	Location
4 bp deletion (TTAG)	3R: 205,032 - 205,035
Single base substitution (A > G)	3R: 206,259
1 bp deletion (T)	3R: 207,180
4 bp deletion (ATTT)	3R: 213,067 - 213,070
7,400 bp insertion (*opus*)	3R: 213,161
Single base substitution (T > A)	3R: 219,883
Single base substitution (G > C)	3R: 220,488
Two base substitution (GC > AA)	3R: 223,222 - 223,223

Reference points

Description	Location
Proximal recombination site	3R: 204,648
tube transcription start site	3R: 213,460
End of *tube* transcription unit	3R: 215,536
Distal recombination site	3R: 223,611
